# Eeg coherence in depressive female adolescents with different types of auto-aggressive behavior

**DOI:** 10.1192/j.eurpsy.2021.2189

**Published:** 2021-08-13

**Authors:** A. Iznak, E. Iznak, E. Damyanovich, I. Oleichik

**Affiliations:** 1 Laboratory Of Neurophysiology, Mental Health Research Centre, Moscow, Russian Federation; 2 Clinical Department Of Endogenous Mental Disorders And Affective States, Mental Health Research Centre, Moscow, Russian Federation

**Keywords:** EEG coherence, Depression, auto-aggressive behavior, female adolescents

## Abstract

**Introduction:**

Intracortical interactions reflected in EEG coherence (Coh) play an important role in the control of behavior in both norm and mental disorders. EEG Coh in depression is less than in the norm. Non-suicidal self-injuries (NSSI) are widespread in adolescents and represent a significant risk factor for consequent suicidal attempts (SA).

**Objectives:**

The aim of the study was to identify the differences in EEG coherence between depressive female adolescents who have NSSI or NSSI and SA in their history compared with healthy controls.

**Methods:**

75 depressive female adolescents (16–25 years old) were enrolled in the study and divided into two subgroups: NSSI (n=38) and NSSI+SA (n=37). The control group included 20 healthy subjects (HC) matched by age and gender. Baseline EEG was recorded, and EEG coherence was analyzed in 8 narrow frequency sub-bands.

**Results:**

In the NSSI subgroup, the number of “high coherent connections” (pairs of EEG leads with Coh>0.80) was the lowest in comparison with the NSSI+SA subgroup (intermediate values) and HC (the highest values) in theta-1 (4-6 Hz), theta-2 (6-8 Hz), alpha-1 (8-9 Hz) and alpha-2 (9-11 Hz) EEG frequency sub-bands, especially in frontal-central-parietal regions.

**Conclusions:**

The lowest EEG coherence in the NSSI subgroup suggests that this subgroup is “more depressive” than the NSSI+SA subgroup, while the NSSI+SA subgroup is “more normal” but has increased suicidal risk. The results obtained suggested the use of EEG Coh data to clarify the degree of suicidal risk in depressive adolescents with different types of auto-aggressive behavior. The study supported by RBRF grant No.20-013-00129а.

**Disclosure:**

No significant relationships.

